# Spatio-Temporal Distribution and Hotspots of Hand, Foot and Mouth Disease (HFMD) in Northern Thailand

**DOI:** 10.3390/ijerph110100312

**Published:** 2013-12-23

**Authors:** Ratchaphon Samphutthanon, Nitin Kumar Tripathi, Sarawut Ninsawat, Raphael Duboz

**Affiliations:** 1 Remote Sensing and Geographic Information Systems Field of Study, School of Engineering and Technology, Asian Institute of Technology, P.O. Box 4, Klong Luang, Pathumthani 12120, Thailand; E-Mails: nitinkt@ait.ac.th (N.K.T.); sarawutn@ait.ac.th (S.N.); 2 Computer Science and Information Management Field of Study, School of Engineering and Technology, Asian Institute of Technology, P.O. Box 4, Klong Luang, Pathumthani 12120, Thailand; E-Mail: raphael@ait.ac.th

**Keywords:** Hand, Foot and Mouth Disease (HFMD), spatial statistics, spatial distribution pattern, temporal distribution pattern, hotspot detection, Geographic Information Systems (GIS), hybrid ring mapping

## Abstract

Hand, Foot and Mouth Disease (HFMD) is an emerging viral disease, and at present, there are no antiviral drugs or vaccines available to control it. Outbreaks have persisted for the past 10 years, particularly in northern Thailand. This study aimed to elucidate the phenomenon of HFMD outbreaks from 2003 to 2012 using general statistics and spatial-temporal analysis employing a GIS-based method. The spatial analysis examined data at the village level to create a map representing the distribution pattern, mean center, standard deviation ellipse and hotspots for each outbreak. A temporal analysis was used to analyze the correlation between monthly case data and meteorological factors. The results indicate that the disease can occur at any time of the year, but appears to peak in the rainy and cold seasons. The distribution of outbreaks exhibited a clustered pattern. Most mean centers and standard deviation ellipses occurred in similar areas. The linear directional mean values of the outbreaks were oriented toward the south. When separated by season, it was found that there was a significant correlation with the direction of the southwest monsoon at the same time. An autocorrelation analysis revealed that hotspots tended to increase even when patient cases subsided. In particular, a new hotspot was found in the recent year in Mae Hong Son province.

## 1. Introduction

**Figure 1:**
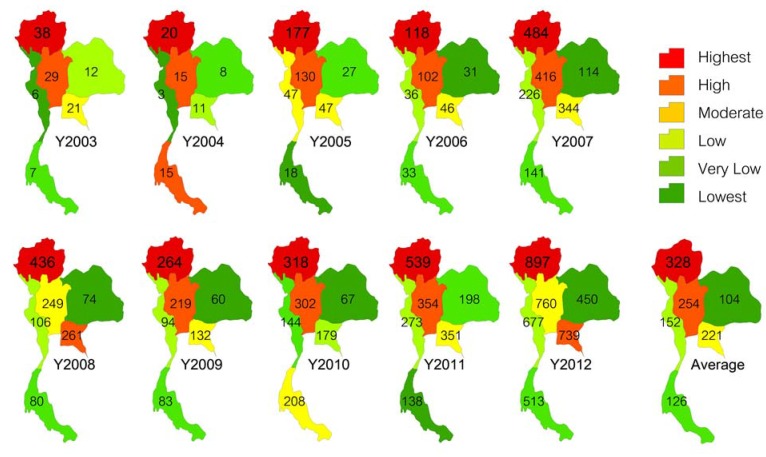
HFMD outbreaks in number of patients per million inhabitants at the regional level, annually from 2003 to 2012 and the 10 year average.

Hand, Foot and Mouth Disease (HFMD) is an emerging illness infecting infants and children. It is characterized by fever, painful sores in the mouth and a rash with blisters on the hands, feet and buttocks. HFMD is most frequently caused by coxsackievirus A16 (CA16) and enterovirus 71 (EV-71) [1,2,3]; however, most patients with fatal complications are infected with EV-71 [4]. At present, no effective chemoprophylaxis or vaccination approaches for dealing with HFMD are available [5,6]. The transmission of HFMD occurs from person to person through direct contact with nasal discharge, saliva or fluid from the blisters. Other infection paths include food or water contaminated with fecal droplets, nasal discharge, fluid or saliva from an infectious person. Weather variables may affect the transmission of HFMD either directly or indirectly [7]. Globally, HFMD outbreaks have been documented for more than four decades. It has been reported that in the last decade the western Pacific region, including countries such as Japan, Malaysia, Singapore, Thailand and China, was the area most severely affected by HFMD [8,9,10,11,12,13]. Other countries, such as Taiwan, Hong Kong, the Republic of Korea, Vietnam, Cambodia, Brunei and Mongolia, were also impacted. HFMD has also progressed to become a leading cause of suffering and mortality in some developing countries, with Thailand finding itself among these. The current epidemic situation in Thailand, as reported by the Bureau of Epidemiology, Ministry of Public Health, includes HFMD outbreaks during the past 10 years, with the more severe in the last 5 to 6 years. Four deaths were caused by HFMD in 2008, and another four were reported in 2009. In 2011 and 2012, six and two deaths were reported, respectively. The northern region of Thailand has had the highest infection rate every year from 2003 to 2012 (Figure 1). 

Statistically, the number of HFMD patients has trended upward for the past 10 years. The number of HFMD cases *per capita* reported by the Epidemiology Center of Thailand reveals that the upper north of Thailand has the highest outbreak rate, with very high infectivity observed in Lampang, Phayao, Nan, Chiang Rai and Lamphun provinces. MThai News reported HFMD outbreaks in many schools in July 2012. Prompted by the situation, the Ministry of Public Health cooperated with the Ministry of Education to establish a war room and to launch a policy to control the outbreak by closing more than 100 schools in several provinces with immediate cleaning and disinfection of the sites. If more than five infected students were detected at a school, the administrator had to close the school for at least 7 to 10 days to prevent further infections and to clean the school. During the same period, there was an outbreak in Cambodia, where more than 60 people died due to HFMD within 3 months. This outbreak was also a major concern for Thailand. The deputy director of the Office of Disease Prevention and Control reported that new species of the virus were found that had not been seen in the area before. It is assumed that the virus could have mutated.

In recent years, several studies have been conducted in different countries to understand the diffusion and transmission patterns of HFMD. However, very few studies have been conducted in Thailand. Moreover, attempts to understand the disease have focused solely on the study of medicine and public health or on its demographic distribution. Thus, a full understanding in terms of the spatial and temporal characteristics of the disease’s transmission pattern has not yet been established. Here, the application of GIS technology may prove useful by allowing for a spatial analysis concerning medical and public health. The assessment of spatio-temporal characteristics and disease associations with weather can provide valuable information for the efficient allocation of public health resources for disease prevention and treatment [14,15,16]. The spatio-temporal features of an infectious disease are usually driven by certain determinants that can provide invaluable information for exploring the risk factors of the disease and contribute to developing effective measures to control and prevent its transmission [17,18]. Spatio-temporal analysis is increasingly used in epidemiological research based on specific or routinely collected data from different sources [19,20,21]. Therefore, a better understanding of the spatio-temporal distribution patterns of HFMD would help in identifying areas and populations at high risk and then formulating and implementing appropriate regional public health intervention strategies to prevent and control the outbreak [22].

The spatial dimensions of HFMD incidence have been explored in view of several explanatory determinants, such as average temperature, average relative humidity and monthly precipitation, to examine possible spatial variations in the assumed relationship between these factors and the incidence of HFMD. Climatic factors have been taken into consideration because they may directly or indirectly affect the transmission of HFMD. HFMD occurs during the summer in temperate regions but at any time in tropical countries [23]. However, there are limited studies that discuss the association between weather and the dynamics of HFMD in Thailand. 

The focus of the present study was to elucidate the spatial and temporal aspects of the incidence of HFMD. The first research objective was to study the general distribution of cases in terms of patient age and gender and the timing of infection. The second objective was to study the spatial distribution of infections, and the third objective was to detect hotspots in the upper north of Thailand from 2003 to 2012. Finally, hybrid ring mapping was applied to better visualize and understand the spatial and temporal domains of the results.

## 2. Materials and Methods

### 2.1. Study Area

**Figure 2:**
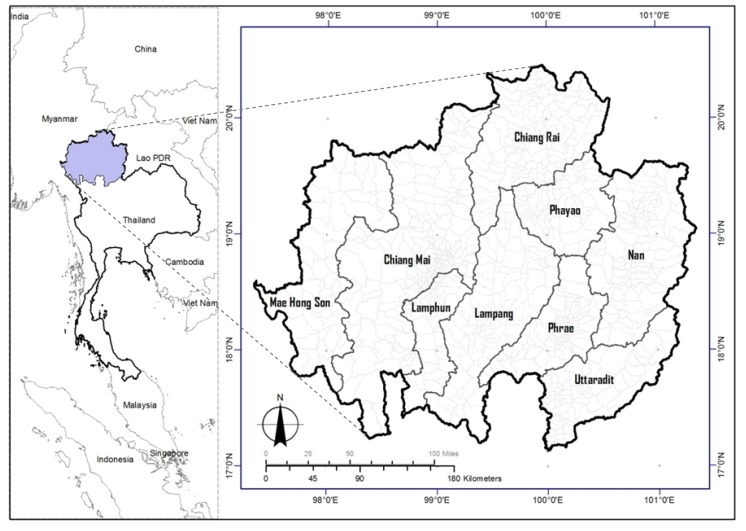
Study area: Upper northern region of Thailand.

Because the upper northern region of Thailand was the area with the highest HFMD infection rate in Thailand over all 10 years from 2003 to 2012, this area was selected as the study area. Geographically, the upper north of Thailand is one of six regions of Thailand and is located between 97°19′8′′E and 101°22′18′′E and 17°11′12′′N and 20°29′1′′N. The region is located approximately 600 kilometers north of Bangkok, covers an area of 93,690.85 km^2^ (9,369 million hectares), or 18.25 percent of Thailand, and has a population of 6,133,208. The area consists of nine provinces: Mae Hong Son, Chiang Mai, Chiang Rai, Lamphun, Lampang, Phayao, Phrae, Nan and Uttraradit. It borders the Republic of the Union of Myanmar to the west and north, the Lao PDR to the north and east and is adjacent to the Tak, Sukhothai and Pitsanulok provinces of Thailand to the south (Figure 2).

### 2.2. Data Acquisition

#### 2.2.1. Disease Data

Disease data were obtained from the Bureau of Epidemiology, National Trustworthy and Competent Authority in Epidemiological Surveillance and Investigation, Ministry of Public Health of Thailand. HDMF case data as reported at the village level from January 2003 to November 2012 were used in this study. The databases comprised the monthly numbers of reported, both apparent and confirmed, HFMD cases in terms of gender, age and the month of the symptoms. 

#### 2.2.2. Meteorological Data

Monthly average temperature (Celsius), monthly total rainfall (millimeters) and monthly relative humidity (percent) for January 2003 to November 2012 were obtained from the nine weather stations operated by the Thai Meteorological Department in each province in the study area. The climate of the upper north of Thailand is subtropical and cooler than the other regions of Thailand. As such, it can be clearly divided into three seasons as follows: summer: February through May; rainy: June through September; and cold: October through January. 

#### 2.2.3. Village Data

The locations and population data for 7,700 villages in the study area were obtained from the Information and Communication Technology Center, Ministry of the Interior, Thailand. To conduct a GIS-based analysis of the spatial distribution of HFMD, points and polygons representing villages from the administrative boundary layer, which were generated based on the administrative boundary map, were obtained from the Geo-Informatics and Space Technology Development Agency of Thailand. All HFMD cases were coded and matched to the village layers by administrative code using ArcGIS 10.0 software. The accuracy of the village point locations was confirmed by overlaying them on satellite images. 

### 2.3. Methodology

#### 2.3.1. Distribution Analysis

First, in the distribution analysis the incidence of HFMD was analyzed by population, area and time using general statistics. The population distribution analysis comprised age group and gender. The temporal pattern of HFMD infection was analyzed at yearly and monthly intervals separately for each province. In addition, the correlation coefficients between HFMD cases and climatic factors such as air temperature, relative humidity and total rainfall from 2003 to 2012 were analyzed for all 10 years by the Pearson method as well as separately for each season. The distribution of HFMD by area was analyzed separately for each province. The annual incidence rates from 2003 to 2012 are also given. 

#### 2.3.2. Spatial Distribution Analysis

A global spatial analysis by spatial autocorrelation was applied to the HFMD incidence rates to analyze their distribution over the study area and to identify spatial disease clusters of statistical significance [19]. The Moran’s I statistical method [24,25] was used to evaluate the spatial autocorrelation in the distribution of HFMD cases and to determine how villages were clustered (random or dispersed) in the area with reference to the HFMD morbidity rate. Village locations and morbidity rates were analyzed for each village. The indices were evaluated by simulation considering the original location of the villages [26] with Moran’s I values ranging from −1 to +1, where a value close to “0” indicates spatial randomness and a positive value denotes a positive spatial autocorrelation and vice versa.

The Average Nearest Neighbor (NN) tool calculates the distance between each featured village point and the nearest neighboring point. The nearest neighbor distances were then averaged. When the average distance was less than one, the distribution of the features being analyzed was considered clustered. An average distance between one and two indicated a random pattern. With an average distance greater than two, the features were considered dispersed. 

The Standard Deviation Ellipse (SDE) measures whether a distribution of features displays a directional trend (whether features are farther from a specified point in one direction than in another). The standard distance circle shows the spatial spread of a set of point locations.

The Mean Center (MC) is the average location of a set of points. Here, the MCs of the locations of the villages having HFMD cases were measured. These indicate the area and extent of the incidence of HFMD cases.

The Linear Directional Mean (LDM) is the line angle representing the mean orientation of all lines in the dataset. The LDM can also measure the trend of their lengths and geographic centers. The output of the LDM is a single line located at the calculated mean center with a length equal to the mean length and an orientation equal to the mean orientation of all input vectors. The mean direction is calculated for features that move from a starting point to an end point. In this case study, the LDM was calculated using the monthly MC movement from January 2003 to 2012. For the future, the LDM trend was calculated as the average LDM of these 10 years. Another LDM was calculated for each season by separating monthly MC movements into seasons (summer, rainy and cold). These LDMs show the LDM trend for each month during the 10 years of the analysis. Moreover, the LDM of each season was explained in relation to the respective monsoon direction.

#### 2.3.3. Hotspot Detection and Analysis

Moran’s I has well-established statistical properties to describe global spatial autocorrelations. However, it is not effective in identifying clustered spatial patterns. These clusters are also described as hotspots [27]. Local indicators of spatial association (LISA) permit the decomposition of global indicators of Moran’s I into the contribution of each individual observation [28]. At the local level, the mean LISA was calculated to identify the pattern as clustered, random or dispersed. This indicator considered both village locations and their attributes. In this case, the attributes were the yearly HFMD morbidity rates, which were also analyzed monthly for each village, and the hotspot locations [28]. The local Moran’s I value was checked at the local level of spatial autocorrelation to identify extreme and geographically homogeneous HFMD morbidity rates [28,29]. The local indicator allowed the identification of HFMD hotspots where the value of the index was extremely pronounced across localities, as well as those of spatial outliers. The simulation used a permutation of the values among neighbors. The significance level was set at 0.05. A Moran scatter plot was created with a standardized HFMD. In this study, the village location points were converted to Thiessen polygons. This follows a definition of neighbors based on common boundaries between polygons. A spatial weights matrix was calculated to define the local neighborhood surrounding each village polygon. This was created to support the spatial autocorrelation measure.

## 3. Results

### 3.1. General Distribution

The trend in the number of patients varied over the analyzed decade, first increasing from 2003 to 2007, then decreasing from 2007 to 2009, and finally reversing with a rapid increase from 2009 to 2012. The highest HFMD infection rates in 10 years were reported in 2012, with 5,500 cases in the study area.

The disease distribution was analyzed based on the age groups of patients. The age groups with the highest frequencies were found in 1- and 2-year-olds, representing 31.81 and 31.05 percent of patients, respectively (Table 1). Most patients were less than 5 years old, accounting for an average of 95.37 percent of all cases. The number of patients rapidly decreased with age. However, it is notable that the trend of patient age changed significantly over the past 10 years, with infections of one-year-olds clearly decreasing while the number of patients aged 2, 4, 5 and 6 years clearly increased.

The ten years of data reveal that the number of male patients was slightly higher than that of females. When separated by age, 1- and 2-year-old males were more likely to be infected than their female peers. In contrast, in children aged 10 years and older, the opposite pattern occurred. Because the majority of patients were aged 0 to 5 years (95.37 percent), the results of only this age group were compared in this study. Male patients accounted for 56.81 percent and females for 43.19 percent of cases. Thus, the ratio of male to female patients was 1.3153 compared with the overall sex ratio for 0- to 5-year-olds of 50.68 percent boys to 49.32 percent girls, or 1.0275 boys per girl. This ratio indicates that boys are more likely to get infected by the virus than girls in the same age group. The overall mean age of male children with HFMD was 2.6 years, which is slightly lower than the mean age for females of 2.8 years. The highest mean age, 3.3 years, was recorded in Nan province. Lampang and Lamphun provinces reported the lowest mean ages of approximately 2.3 years. Patients have been reported from every province, ranging from infants to the elderly. Chiang Mai recorded the oldest patient, aged 78. For the period from 2003 to 2012, Chiang Rai and Lampang provinces had the highest average occurrences of HFMD, with infection rates of 22.82 and 20.30 cases per 100,000 inhabitants, respectively.

Looking at the average monthly infections over ten years, the month of highest HFMD infection appears to be June, with 12.69 percent of all infections, followed by July with 11.92 percent, both of occurring during the rainy season, whereas November, with 11.02 percent, is in the cold season. The lowest incidence occurred in April, with 2.65 percent, followed by March, with 4.40 percent, both belonging to the summer period.

**Table 1:** Distribution characteristics of HFMD by population, area and time, 2003–2012.

	2003	2004	2005	2006	2007	2008	2009	2010	2011	2012	%
**Age**	**(Percentage)**										
<1	10.50	8.26	6.65	7.50	4.95	6.98	4.95	5.79	6.05	11.60	7.55
1	35.71	45.45	34.61	36.29	31.68	31.79	30.22	29.66	30.56	32.25	31.81
2	23.53	19.83	31.51	29.47	33.81	30.65	31.52	32.27	32.41	29.04	31.05
3	15.13	12.40	16.67	12.28	17.09	16.99	17.89	15.32	18.14	13.55	15.93
4	3.78	4.96	5.37	6.68	6.52	6.76	7.00	7.53	6.41	5.89	6.40
5	1.26	1.65	1.91	2.32	2.26	2.44	3.22	2.87	2.84	2.78	2.62
6	1.26	0.83	0.64	0.68	1.30	1.07	1.42	1.43	1.27	1.75	1.35
7	0.42	0.83	0.36	1.09	0.57	0.85	0.62	1.02	0.73	0.78	0.74
8	0.84	0.83	0.55	0.14	0.27	0.52	0.74	0.61	0.30	0.53	0.47
9	0.42	0.83	0.46	0.82	0.23	0.33	0.37	0.15	0.39	0.33	0.34
10	0.00	1.65	0.46	0.27	0.40	0.41	0.37	0.36	0.24	0.31	0.35
>10	7.14	2.48	0.82	2.46	0.93	1.22	1.67	2.97	0.67	1.20	1.39
**Gender**	**(Number)**										
male	140	70	629	414	1,712	1,484	922	1,112	1,909	3,130	56.81
female	98	51	469	319	1,296	1,224	693	840	1,399	2,370	43.19
total	238	121	1,098	733	3,008	2,708	1,615	1,952	3,308	5,500	100
**Province**	**(Ratio per 100,000 Inhabitats)**										
Chiang Mai	0.75	0.12	6.12	3.50	17.78	28.74	9.37	15.24	34.44	80.73	16.01
Chiang Rai	0.49	0.99	10.37	13.22	36.90	56.71	28.37	50.58	59.48	126.39	22.82
Lampang	17.56	8.86	62.96	26.75	115.49	81.81	50.88	35.17	79.60	57.42	20.30
Lamphun	10.02	0.74	11.61	10.11	105.14	42.70	26.93	18.29	37.88	42.58	6.11
Mae Hong Son	5.04	1.23	32.33	9.01	17.66	18.60	17.37	20.19	61.87	194.63	4.58
Nan	0.62	1.05	24.89	23.66	76.67	29.62	54.04	57.52	94.42	81.62	10.44
Phayao	2.19	4.10	16.02	12.13	46.86	64.01	31.61	36.81	65.99	147.59	10.26
Phrae	0.62	0.21	9.33	10.46	29.41	26.32	17.31	31.25	41.20	35.10	4.59
Uttaradit	2.08	1.28	2.34	4.49	36.11	23.48	19.87	23.35	34.92	66.37	4.89
**Month**	**(Number)**										
January	5	46	7	48	33	963	28	309	59	387	9.29
February	3	17	11	56	19	367	53	393	35	380	6.58
March	4	22	7	43	12	157	96	247	40	264	4.40
April	4	4	13	43	8	80	36	90	42	218	2.65
May	5	9	152	81	31	195	97	179	149	405	6.42
June	11	5	329	78	78	320	268	209	466	809	12.69
July	21	3	196	79	134	173	146	134	345	1187	11.92
August	13	8	98	74	117	176	138	112	324	620	8.28
September	24	2	94	90	202	96	209	85	506	514	8.98
October	45	2	79	67	386	69	172	59	444	443	8.71
November	39	1	77	47	980	63	198	68	488	273	11.02
December	64	2	35	27	1008	49	174	67	410	0	9.05

### 3.2. Temporal Distribution Pattern

**Figure 3:**
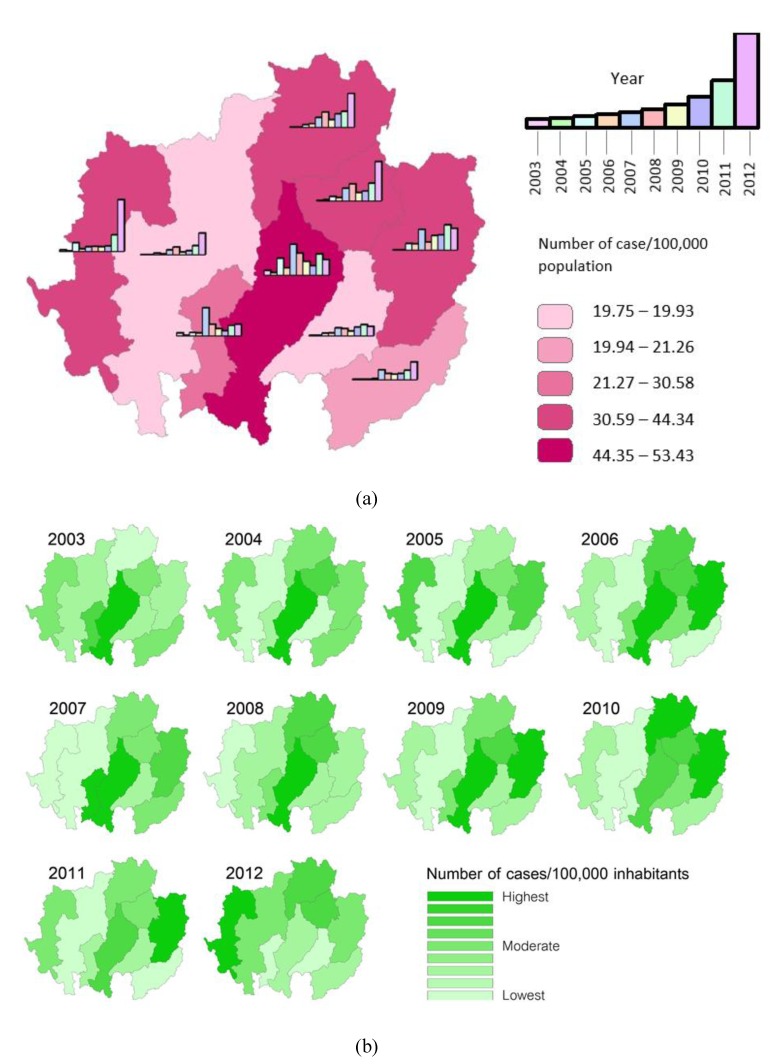
(**a**) Bar charts of the HFMD infection rate for each year by province, shown as average case counts for 2003–2012 per 100,000 inhabitants. (**b**) Yearly Ranking of HFMD cases *per capita*.

The investigation of the temporal distribution of infections in each province from 2003 to 2012 revealed that most maxima in terms of the number of patients occurred in the latter part of the decade, mostly in 2011 and 2012, with the exception of Lamphun and Lampang provinces, where the highest patient case counts occurred in 2007. It is notable that some outbreaks in neighboring areas had similar temporal patterns. In Phrae and Nan, outbreaks occurred during the same period in 2011, and likewise, Chiang Rai and Phayao saw an outbreak peak in 2012 (Figure 3a). 

Figure 3b shows the rates of HFMD cases *per capita* for each year. The annual disease incidence rate was highest in Lampang province, located in the central part of the study area, from 2003 to 2008. The area with the highest incidence rate then moved east to Nan from 2009 to 2011. In 2012, the highest incidence rate was recorded in Mae Hong Son province, located on the western side of the study area.

**Figure 4:**
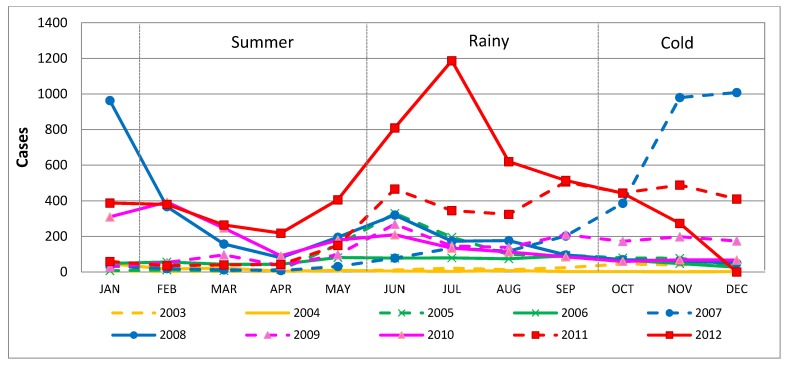
Monthly HFMD cases from 2003 to 2012.

The monthly reported cases of HFMD are summarized in Figure 4. The highest peaks of infections per year occurred in different months. However, most outbreaks occurred during the rainy and cold seasons, except for 2010, when a peak occurred in February. Over the entire investigated period, April was the month with the fewest detected outbreaks, particularly in 2006 and 2007.

**Figure 5:**
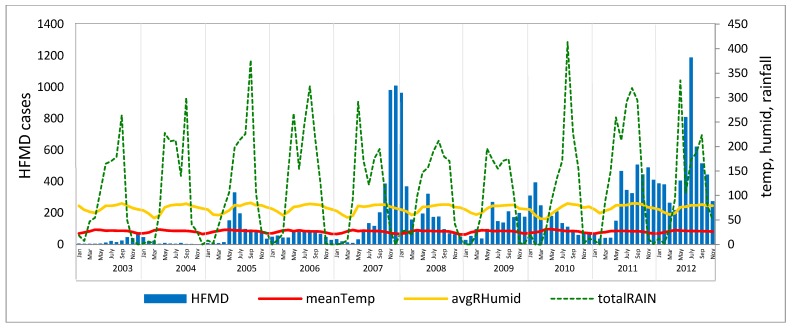
HFMD case counts per month from 2003 to 2012 related to climatic factors.

The HFMD outbreaks do not directly correlate to the annual seasonal cycle. The temporal outbreak pattern can be described as five waves with a wave occurring every two years (Figure 5). However, the analysis of the correlation between climatic factors and HFMD incidence in terms of Pearson’s correlation coefficient revealed that the overall annual average temperature had a low negative relationship with an HFMD incidence of 0.123, whereas relative humidity and total rainfall exhibited low positive relationships with HFMD of 0.166 and 0.045, respectively**.**

When analyzed by season, the summer periods were consistent across the 10 years. Temperature had a negative correlation with disease incidence in six of the 10 years, humidity had a positive correlation in 9 of the 10 years and rainfall a highly positive correlation in seven of the 10 years. The rainy season exhibited a significant difference in relationship, but no clear relationship was detected for the cold season (Table 2). These results indicate that lower temperatures and higher humidity may cause an increase in outbreaks in the summer season. In contrast, during the rainy season, increased outbreaks appear to be associated with higher temperatures and lower humidity.

**Table 2:** Pearson’s correlations between climatic factors and HFMD infection rates by season.

	Summer			Rainy Season			Cold Season		
year	Temperature	Humidity	Rainfall	Temperature	Humidity	Rainfall	Temperature	Humidity	Rainfall
2003	+0.808	+0.015	+0.992	+0.068	+0.601	+0.721	+0.167	−0.688	−0.171
2004	−0.561	−0.433	−0.575	+0.840	−0.532	−0.912	−0.326	−0.834	−0.518
2005	+0.602	+0.942	+0.762	+0.881	−0.705	−0.628	+0.899	+0.990	+0.696
2006	−0.554	+0.905	+0.693	−0.221	+0.069	−0.388	+0.939	+0.891	+0.808
2007	−0.305	+0.925	+0.734	−0.814	+0.740	+0.317	+0.017	+0.350	−0.174
2008	−0.980	+0.241	−0.383	+0.976	−0.940	−0.566	−0.319	−0.765	−0.316
2009	+0.005	+0.302	+0.431	−0.347	−0.084	+0.586	+0.663	+0.439	+0.269
2010	−0.941	+0.333	−0.544	+0.949	−0.940	−0.562	−0.267	−0.838	−0.277
2011	+0.623	+0.887	+0.857	+0.160	−0.003	−0.528	+0.589	+0.673	+0.321
2012	−0.481	+0.742	+0.460	−0.171	−0.286	−0.398	−0.113	−0.193	+0.280

### 3.3. Spatial Distribution Pattern

**Figure 6:**
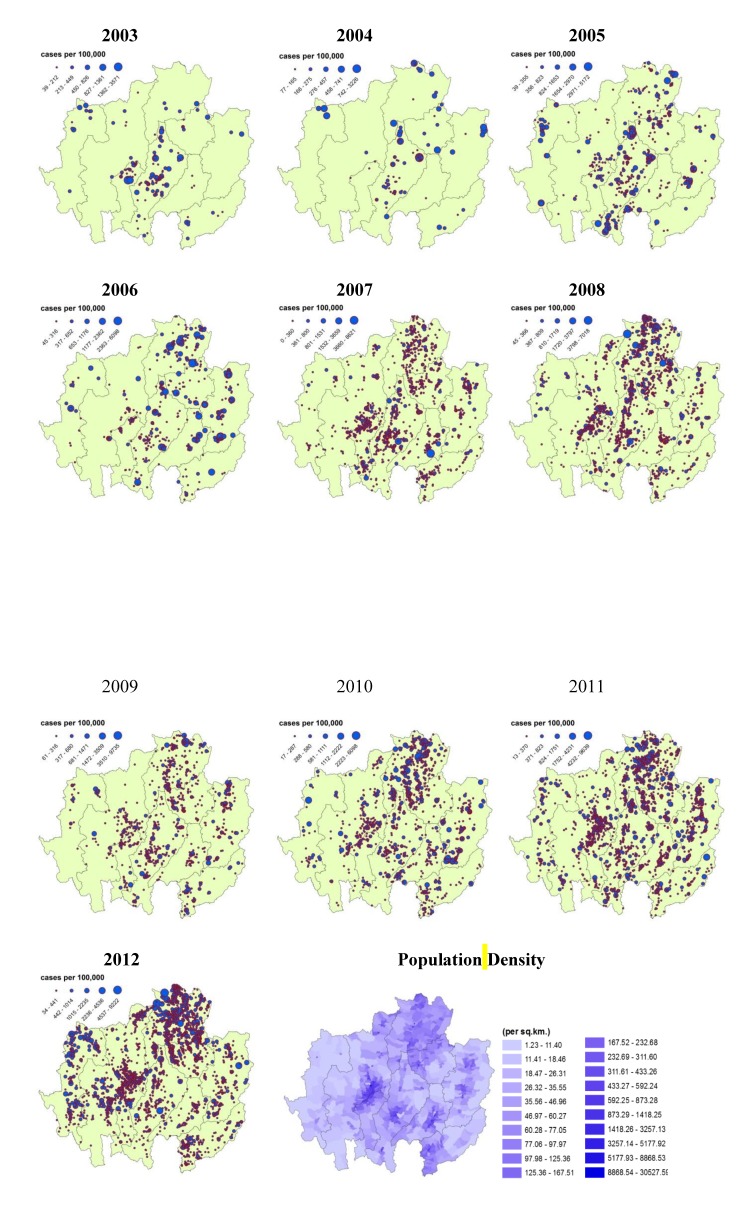
Annual HFMD incidence rates (2003–2012) and population density of the provinces in the study area in Northern Thailand.

The HFMD-affected villages were classified based on the Jenks natural breaks optimization method. Figure 6 presents the annual incidence rates from 2003 to 2012. In 2003, the villages with high incidence rates were mostly found in the center of the study area (Lamphun and Lampang provinces). Later, however, the highest rates spread toward the east, particularly into the north-south belt (Chiang Rai and Lampang provinces) and Nan province in 2007. After that, a general spread across the study area can be observed. In recent years, outbreaks were observed in three main groups, located in Chiang Rai-Phayao and the central parts of Chiang Mai and Mae Hong Son provinces.

The population density map displays the high densities of the larger municipal areas in every province except Mae Hong Son, which has no clear cluster. The comparison of the annual HFMD incidence maps and the population density map indicates a correlation. In general, high HFMD incidence rates were found in areas with a high population density except for Mae Hong Son province, where many patient cases occurred, particularly in 2012.

### 3.4. Moran’s I

The HFMD distribution patterns at the village level were established by analyzing the raw infection rate (patient cases per unit population) in terms of spatial autocorrelation (Moran’s I). The results of the Moran’s I analysis presented values higher than zero for almost every year. Table 3 shows that most years exhibited a clustered pattern except for 2007, which displayed a dispersed pattern. All years showed different levels of clustering; 2004 had the highest values for both the Moran’s I and Z scores (0.634 and 8.645, respectively), with most groups located in lower-central Lampang province (Khokha and MaeTha districts). The second and third highest values were observed in 2005 and 2010, respectively. 

Although most annual distribution values were low, when separated by season very prominent values were detected for the rainy season, particularly in 2006, 2007 and 2009 through 2012. For the cold season, the majority of cluster patterns were found in 2009, followed by 2012 and 2006. For the summer season, which generally exhibited only low numbers of affected villages, the most clustered pattern was found in some areas in 2004.

### 3.5. Mean Center (MC), Standard Deviation Ellipse (SDE) and Linear Directional Mean (LDM)

**Figure 7:**
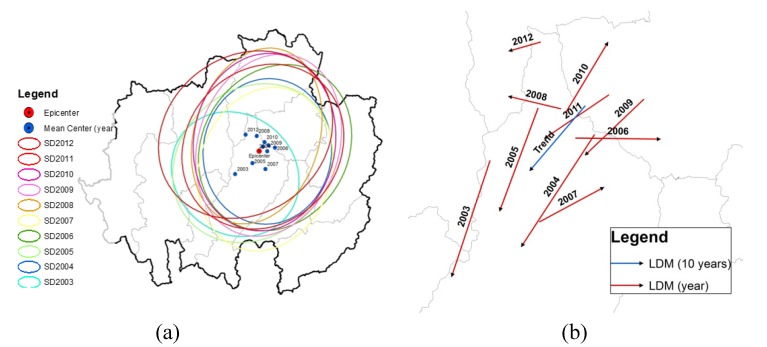
(**a**) HFMD Mean Center and Standard Deviation Ellipse and (**b**) Linear Directional Mean from 2003 to 2012.

The analysis of the annual spatial MC revealed that most clusters occurred in the central portion of the study area in the north of Lampang province. The MC for 2003 was in a different location and was far less pronounced compared with the other years. The SDE of 2003 was the smallest, falling within the main area of Lampang and Lamphun provinces. Most SDEs appeared at the same location with the same shape, size and northeast-southwest direction during 2004 to 2012. They appeared primarily in 7 of the 9 provinces, namely in Chiang Mai, Chiang Rai, Lamphun, Lampang, Phayao, Phrae and Nan provinces (Figure 7a).

Before producing the yearly diffusion of the disease as shown in Figure 7b, the movement of the monthly MC location was examined. It was found that most LDMs were directed toward the south, except in 2006, 2007 and 2010. However, the LDM trend displayed a northeast-southwest direction with an orientation of 220 degrees. This result indicates the global trend of HFMD spread in the study area.

**Table 3:** Annual and seasonal global spatial autocorrelation analysis of HFMD.

Year	Moran	Zscore	Pattern	Summer			Rainy Season			Cold Season		
				Moran	Zscore	Pattern	Moran	Zscore	Pattern	Moran	Zscore	Pattern
**2003**	0.033	1.345	C	−0.12	−0.19	D	1.15	3.03	C	2.66	5.00	C
**2004**	0.634	8.645	C	8.76	16.06	C	−0.14	−0.71	D	0.14	1.22	C
**2005**	0.161	0.161	C	5.88	11.23	C	2.11	15.21	C	2.32	9.36	C
**2006**	0.098	0.189	C	1.20	3.72	C	6.50	34.75	C	4.20	13.58	C
**2007**	−0.002	−0.006	D	5.26	7.99	C	5.27	31.25	C	3.19	56.88	C
**2008**	0.032	0.162	C	7.58	64.67	C	1.82	13.61	C	2.50	16.52	C
**2009**	0.082	0.335	C	0.18	0.75	C	3.82	31.69	C	7.02	35.51	C
**2010**	0.108	8.073	C	1.77	13.76	C	4.08	25.35	C	0.77	3.74	C
**2011**	0.040	0.202	C	0.33	1.05	C	3.14	41.27	C	0.55	6.94	C
**2012**	0.026	0.179	C	6.06	68.35	C	4.59	105.06	C	4.96	40.80	C

Note: C = Clustered pattern; R = Random pattern; D = Dispersed pattern.

### 3.6. Hotspot Detection and Analysis

**Figure 8:**
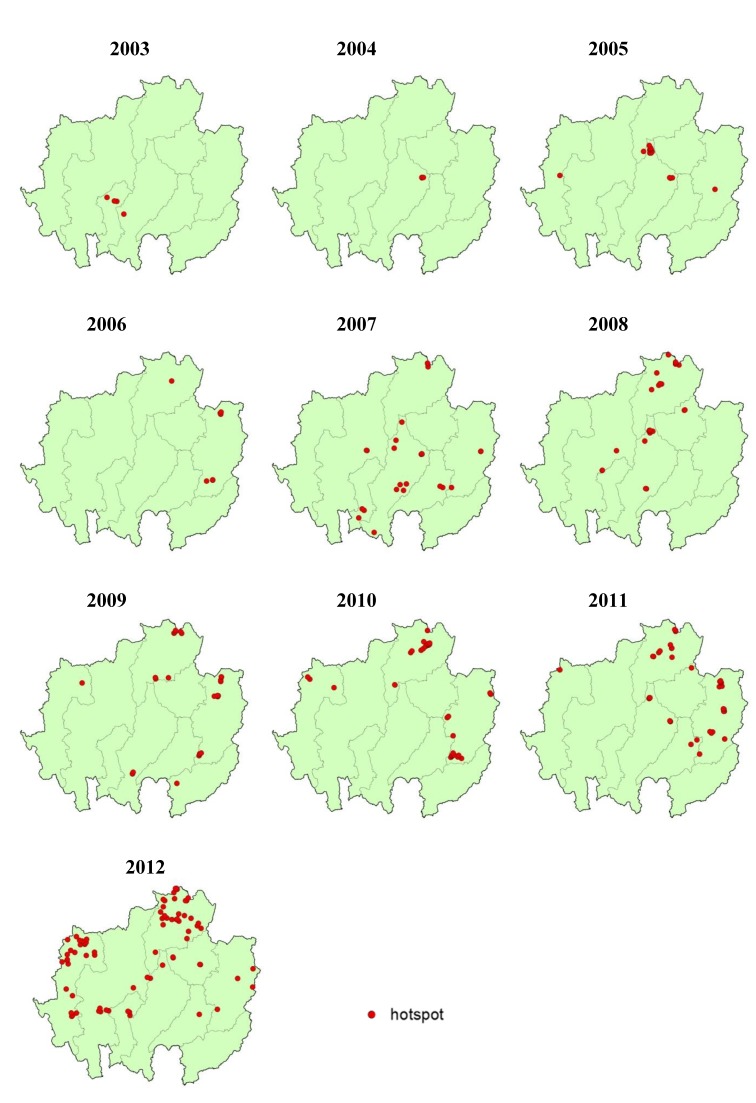
HFMD hotspot development from 2003 to 2012.

Hotspot detection means the identification of high-HFMD-incidence villages that are surrounded by other villages with a high HFMD incidence. The result of the Moran’s I scatter plot illustrates the spatial autocorrelation of HFMD incidences at the village level with a significant Moran’s I value (*p*-value < 0.05). The number of hotspots increased from 2003 to 2012, spreading from their origin in the lower central region of the study area in 2003 (Lamphun and Lampang provinces, Figure 8). In 2004, the hotspot-containing area moved to northern Lampang province. Then, 2005 saw the area move to the border area of Lampang and Chiang Rai provinces. In 2006, hotspots emerged in the eastern and northeastern margins of Nan and Chiang Rai provinces. In 2007, hotspots appeared to have scattered across the entire area except Mae Hong Son, Phayao and Uttaradit provinces. In 2008, the hotspot-containing area was found to be smaller than during the previous year, limited to the central and northern part of the study area. In 2009, hotspots spread to the margin areas again, and in 2010, a similar development followed in Chiang Rai, Mae Hong Son and Nan provinces. In 2011, distinct hotspots were observed clustered in the east and the north of the study area, and in 2012, most hotspots were located in the north and west of the study area.

**Figure 9:**
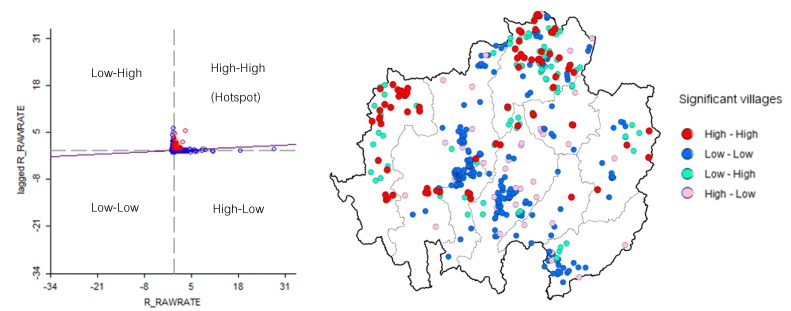
Moran’s I scatter plot matrix and LISA cluster map of HFMD for 2012.

The highest number of hotspots was observed in the last year, 2012 (Figure 9), totaling 75 villages, which can be divided into two groups: those in the northern part of Chiang Rai province and those at the northern tip of Mae Hong Son province. With 29 hotspots, Chiang Rai had the highest count, followed by Mae Hong Son with 25. Classifications by month illustrate the hotspots’ development during this year. No hotspots were found in Uttaradit province. It can be noted that although many villages were significantly affected in Chiang Mai, Lampang and Uttaradit provinces, the hotspots in those areas disappeared. Numerous hotspots were only observed in Chiang Rai and Mae Hong Son province.

There was a significant local Moran’s I as classified by association. The red (high-high) and blue points (low-low) are indicators of spatial clusters, opposite the cyan (low-high) and pink points (high-low), which are indicators of spatial outliers. The meanings of red, blue, cyan and pink are as follows: high infection rate surrounded by high rates, low surrounded by low, low surrounded by high and high surrounded by low, respectively.

**Figure 10:**
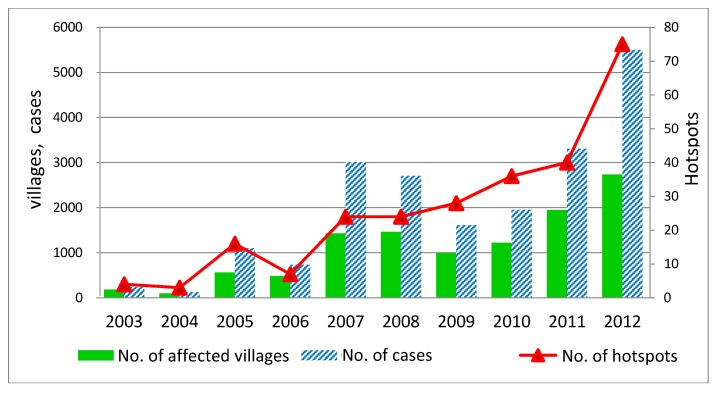
Number of affected villages, number of cases and number of hotspots in the upper north of Thailand during 2003–2012.

Figure 10 shows the changes in the number of affected villages, the number of cases and the number of hotspots from 2003 to 2012. The number of affected villages followed a similar trend to that of the number of cases over nearly the entire period. In contrast, the trend in the number of hotspots differed, especially in the period from 2007 to 2012. After 2007, the number of affected villages and HFMD cases decreased, whereas the number of hotspots increased continuously until 2012.

The line graph confirms that the trend in HFMD outbreaks is more severe. In particular, the new outbreak in the northern part of Mae Hong Son province (Mueang, Pangmapha and Pai districts) should be closely watched to prevent HFMD outbreaks in the future.

**Figure 11:**
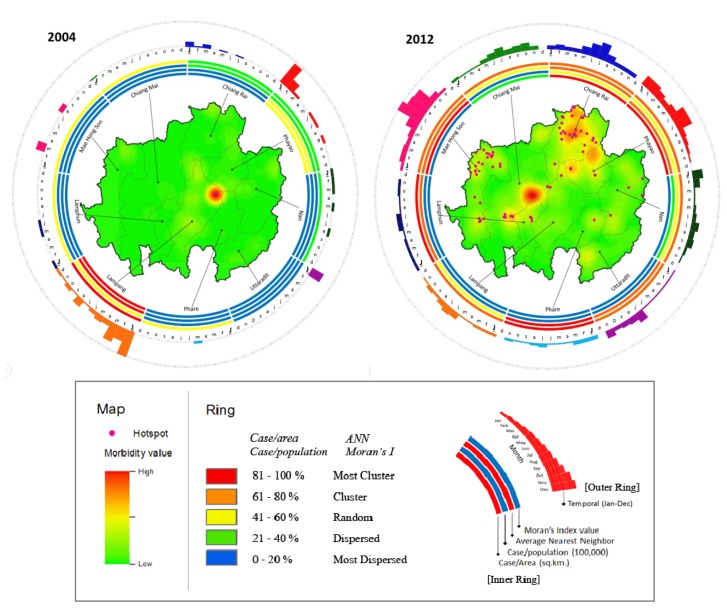
Spatio-temporal ring map: The inner ring shows the spatial distribution of hotspots and morbidity; the outer ring shows the temporal distribution.

The Hybrid ring maps illustrate the combination of the results of the spatial and temporal analyses. Ring mapping aids in better understanding HFMD outbreak patterns in both geographical and temporal terms. In addition, the map of centers, also called a morbidity map, which is derived from the kernel density method, can illustrate the number of patient cases. The map also displays hotspots derived by the LISA method, which answers the question of “how to”. Moreover, the rings visually display the result of the spatial distribution analysis of four values: patient cases per area, patient cases per capita, NN and Moran’s I value. This was carried out for every year from 2003 to 2012, but for simplicity, only the ring maps showing the highest (2012) and lowest (2004) HFMD incidences were included in Figure 11.

The Hybrid Ring Maps for both years represent clear differences in spatial and temporal results. Although the smallest outbreak was observed in 2004, interestingly, both hotspots and high morbidity can be observed in the same area, located in the northern part of Lampang province. The inner ring showed more clustering (81%–100%) in this province than in the surrounding provinces. The monthly variation in outbreaks (outer ring) was also higher than in the other provinces. However, as represented by the outer ring of 2004, several other provinces, including Phayao, Uttaradit and Lampang, also saw many outbreaks in January.

Among the 10 years analyzed, 2012 included the highest number of outbreaks, as shown in Figure 11. The highest morbidity was observed in the central regions of Chiang Mai (Mueang, Sansai and Doisaket districts) and Chiang Rai (Mueang district) provinces. Hotspots were found clustered in the northern part of Chiang Rai province and the north of Mae Hong Son province. However, the highest spatial density was observed in Chiang Rai and Phayao provinces (showed in inner ring). Although no high-morbidity area was observed in Mae Hong Son province, many hotspots (appearing in the Mueang and Pangmapha districts) and high patient case numbers per capita (second inner ring) were observed. The Moran’s I and NN values for this province were also high. In 2012, the 3rd and 4th inner rings showed the highest clustering in Phrae province, although fewer hotspots and lower morbidity were seen in this province. In this year, most provinces, including Mae Hong Son, Chiang Rai and Phayao, exhibited a clear increase in outbreaks during June and August (rainy season).

## 4. Discussion

HFMD has recently become a health issue in Thailand. Most outbreaks occur in the northern region, which was chosen as the study area, and are likely to intensify, as 2012 in particular saw a sharp and unprecedented increase in reported cases. Knowledge and research on this disease has been limited regarding epidemiology and public health. The results reported in this article are different from those limited to statistical distributions and spatial analysis. GIS was employed to enhance the understanding of distribution patterns in the study area. Although GIS has been used in medical and health applications in Thailand for more than 10 years, it appears not to have been applied to studies of HFMD until now, while the number of patients has been increasing rapidly. This report introduced Ring Mapping to improve the visualization of the results. This may be an advantage not only for medical and public health organizations but also for the general public in the affected area to further understanding of the disease and the necessity for its surveillance. 

The results revealed that most patients were infants and children under the age of 5 years, which corresponds to the studies conducted in other countries [4,5,8,9,11,22,30,31,32,33,34,35,36,37,38]. According to seroepidemiological studies [39,40], more than 50% of children under the age of 5 years lack neutralizing antibodies against EV71 and CA16. Additionally, in a previous study, the levels of circulating antibodies had waned so rapidly only 1 month after birth that none of the infants tested had maternal antibodies to EV71 [12]. A recent seroepidemiological study showed that the level of maternal antibody titers declined markedly during the first 7 months after birth, then increased significantly from month 12 to months 27–38. This explains why the majority of patients belonged to the age group of 1- to 2-year-olds, with the incidence rate decreasing with increasing age. The results confirmed that the peak incidence occurs in the 1 year age group [4,5]. However, the infection rate is still likely to increase in the 2-, 4- and 6-year-old age groups and thus might shift from nurseries to kindergartens and schools, where children are more exposed to infection risk by intensive contact and activities with peers. These results reflect those of a study conducted in Hong Kong that found that the number of older children (>5 years) infected increased from 25.4% in 2001 to 33.0% in 2009 [37]. 

Outbreaks were found to affect males more than females, which is in line with previous studies [4,5,38] that found that boys were more susceptible to enterovirus than girls. [38]. Boys may be more physically active than girls, which might lead to more physical contact, promoting the spread of HFMD [4]. The results presented here also indicate that the patient sex ratio (male:female) was higher than the population sex ratio. Remarkably, the number of female patients aged 10 years or older was higher than the number of males of the same age. The reasons for the higher infection and disease outbreak risk in children might be that they are less likely to have developed protective antibodies than adults [18]. Moreover, many children of this age are attending nursery school or kindergarten, where they stay together with their peers of the same age group.

The temporal distribution over the 10-year study period revealed an outbreak pattern recurring every two years, with the largest wave observed between 2011 and 2012, whereas in Sarawak, HFMD outbreaks are reported to occur every three years [23]. Looking at the results by province, the peak outbreak at the end of the decade, in 2011 and 2012, was observed in all provinces with the exception of Lampang and Lamphun provinces, which saw a peak outbreak in 2007. The endemic source analysis investigating the annual outbreak focuses found most of them in Lampang province. Both results match significantly. It is possible that Lampang, after facing the largest and longest outbreak in the past, is reaching the end of the epidemic cycle.

The distribution classified by season found that HFMD appeared primarily during the rainy season and in winter, similar to its occurrence in Yunnan in the south of China, where outbreak peaks were observed in May as well as winter [41]. In contrast, April, during the Thai summer, showed HFMD incidence rates that clearly decreased to the lowest level almost every year. A correlation analysis between HFMD cases and temperature over a long-term period showed a negative result. This contrasts with the results of a study conducted in China, Malaysia, Taiwan, Japan and England that recorded a trend toward peaks in HFMD cases in April [3,4,8,22,23,42]. A study in Belgium found that HFMD infections usually occur throughout the year [43]. In Hong Kong, HFMD occurrence is reported to peak in winter, perhaps due to an increase in winter temperature [37]. Both results showed positive relationships with humidity and rainfall. The reason for the increase in outbreaks in the rainy season may be a weakened immune defense system, especially in children. On the other hand, cooler weather may be associated with behavioral patterns that lead to increased contact among children, e.g., sharing toys and other items in child-care centers or kindergartens, thereby contributing to the spread of EV71 infections [12]. In summer, the negative correlation result may be due to higher temperatures that may destroy or hinder the growth of the virus. The long school holiday in summer may also contribute to the low incidence rate. Most children stay at home, so there is less physical interaction with their peers than at school. Although the exact mechanism is unclear, we can conclude that weather factors have a significant influence on the HFMD infection rate [7,34]. HFMD does occur in temperate countries during summer, but in the tropical countries, it occurs throughout the year [7,23,44,45]. In contrast, the results of a previous study conducted in Tokyo, Japan indicated that higher temperature and humidity may have stimulated the increased HFMD incidence observed between 1999 and 2002 [13].

**Figure 12:**
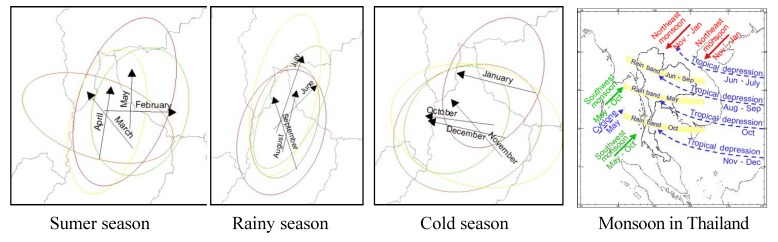
Linear directional mean for each season and the annual monsoon direction in Thailand.

GIS and a global spatial analysis method with spatial autocorrelation were applied to study the occurrence of the disease at the village level over the study period from 2003 to 2012. The results showed that the distribution patterns were clustered throughout all years except 2007, during which the cases exhibited a dispersed pattern. The most intense clustering occurred in 2004, with a Moran’s I of 0.634. Viewed by season, the most prominent outbreaks were found during the rainy and cold seasons. The mean center site remained in the northern part of Lampang province over the whole period. The Standard Deviation Ellipses of the villages affected by the disease covered nearly the entire area in the middle north and the northeast with seven out of all nine provinces. The trend of the global diffusion pattern was from northeast to southwest. The Linear Directional Mean classified by season revealed the influence of the annual monsoon (Figure 12). Significant differences occurred between the individual rainy seasons, but all LDMs followed a similar path in the northern direction. This corresponds to the southwest monsoon during this time of the year, and it indicates that the movement of the MC during this period is influenced by the southwest monsoon. This is consistent with studies conducted in China reporting that from May to June, intense disease clusters began to move from the south to the north [46]. The LDMs in the summer months showed different, irregular patterns. The monthly LDMs of the cold season, even though not clearly associated with the northeast monsoon, showed significantly similar patterns.

Hotspot detection is useful in finding a local cluster. The results indicated that the starting points lie in high morbidity areas. However, in the last year of the study period, they were found even in areas of low morbidity, especially in the west of the study area. Hotspots were found in two main groups in Chiang Rai and Mae Hong Son provinces, with only sporadic hotspots in other provinces. The hotspots were not associated with the number of patients because their number does not decrease along with the number of patients. This means that the probability that a village surrounded by other villages infected with HFMD is a hotspot increases exponentially. The results indicate that the trend of severe HFMD outbreaks has sharply increased from 2003 to 2012. Therefore, all agencies are urged to take measures to prevent outbreaks of this disease.

## 5. Conclusions

This study aimed at understanding the phenomenon of HFMD outbreaks and their spatio-temporal patterns in the upper north of Thailand from 2003 to 2012 using GIS tools. The demographic analysis of patients revealed some interesting facts. It was found that 95.37% of cases were observed in children less than 5 years old. Female children in this age group were less affected than males. Studies in other countries reported similar findings. The male to female ratio of patient cases was 1.32. Cases in children less than one year of age occur much less frequently. Children in the 4–6 age groups are most vulnerable. There are clear indications that children attending kindergarten are more susceptible to HFMD; this may be vital information for public health officers seeking to control the spread of this disease.

The trends of outbreaks and hotspots were also investigated using maps and graphs. Spatially distributed clusters were observed in different provinces in every year except 2007. It was also revealed that the number of hotspots is rising, though the number of cases is slightly declining. This clearly indicates the severity of this disease in new locations, which is alarming if proper measures are not taken to control outbreaks. As revealed by the LDM analysis of different seasons, the diffusion of outbreaks during the rainy season was higher, moving from the southwest toward the northeast, which coincides with the direction of the annual southwest monsoon. 

The temporal analysis on a monthly basis revealed that outbreaks occurred approximately every two years, mostly during the rainy and cold seasons. This implies a significant correlation of HFMD incidence and climatic factors. In the future, this finding may be further consolidated by including more climatic factors and variability. From this research, it can be concluded that GIS proved to be a powerful tool in monitoring HFMD outbreak patterns both spatially and temporally. The results may be utilized by public health officials and the general public to spread greater awareness and to take effective measures to prevent the disease.
